# Editorial Notes

**Published:** 1922-09

**Authors:** 


### Editorial Notes.

**The New Vice-Chancellor.**

Mr. T. Loveday, M.A., has taken up his duties as Vice-Chancellor of the University this session. His personality, no less than his record of work accomplished elsewhere, assures him a cordial welcome.

**"Insulin" in the Treatment of Diabetes.**

A striking advance in the therapeutics of diabetes has been made by Professor J. J. R. Macleod of Toronto. Ever since his book on *Physiology and Bio-chemistry in Modern Medicine* first appeared in 1918 it has filled in a fashion different from any other book on physiology a want which every physician must have experienced. Professor Macleod has brought physiology where we all wanted it most, namely to the bedside of the patient.

The treatment of diabetes has been receiving much attention lately. The fasting method first suggested by Guelpa and revived by Allen seemed for the time being to be holding the field in England and America, though French observers were less enthusiastic in its praise. But even those of us who employed the Allen regime did so with the conviction that at best it only enabled the patient to bend before the storm. Diabetic patients could not be said to be "cured" by it in the sense of having their malady removed.

Now, however, Professor Macleod offers a remedy which may prove a " cure " in a true sense as thyroid extract cures myxœdema. He does not claim that the tests are complete, but so far as the investigations have gone the results have proved so remarkable that no apology is required for alluding to them.

Starting from the standpoint that the inability of the tissues of the body to deal with the excess of sugar in the blood characteristic of true diabetes depended on a deficiency of an unknown internal secretion of those specialised cells of the pancreas found in the islets of Langerhans, Professor Macleod has obtained a pancreatic extract which contains the products of the islets without the products of the other portions of the pancreas.

The method at first adopted consisted in ligating the pancreatic duct in the dog and allowing the dog to survive the operation for seven or ten weeks. After this interval the dog was killed and the pancreas found to have degenerated entirely so far as its acinous cells, whilst the islets of Langerhans remained active. An extract of such a pancreas if injected intravenously into pancreatectomised and therefore diabetic animals reduces the blood sugar and the amount of sugar in the urine of such animals, enabling them to live far beyond the usual period.

In later experiments it was found that the pancreas of fœtal calves resembles that produced by ligation of the duct, in that it contains none of the proteolytic ferments of the acinous cells, and to all intents may be considered as yielding only the products of the islets. By extracting such fœtal pancreatic tissue a highly potent and readily procurable preparation may be obtained.

This extract Professor Macleod and his able collaborators have named " Insulin." It has been tested upon artificially " diabetic " animals, with the results mentioned. But further trials made on human subjects showed parallel results. Patients suffering from diabetes treated by means of intravenous injections of "Insulin" report complete relief from the subjective symptoms of their disease; the blood sugar falls, the respiratory quotient rises, indicating carbohydrate utilisation; the urine sugar falls even to the point of disappearance; whilst ketonuria is abolished.

Evidently in this extract we have a therapeutic measure of unquestionable value in the treatment of certain phases of diabetes in man.

Professor Macleod has favoured us with his latest publications on this important work, and he adds in a covering letter, "We are doing everything in our power to hurry along the production of "Insulin" for therapeutic purposes, and with this end in view are collaborating with the Medical Research Council of England."

Professor Macleod concludes his letter with the promise that he will send to this Journal further information as to how the work is proceeding.

The Editor of this Journal in 1893, nearly thirty years ago, concluded from his investigations at the Bristol Royal Infirmary that "practically nothing can be expected from diabetes treated with pancreatic extract by the mouth." Convinced that it was the internal secretion of the pancreas which must therefore contain the essential substance lacking in pancreatic diabetes, he resorted to grafting freshly-killed sheep's pancreas in a diabete, and further had arranged for obtaining serum from blood to be collected from the pancreatic vein of a living animal, when Dr. Leonard, after promising to assist, rightly declined until a "licence" was obtained. A vivisection licence was a difficulty not easily overcome here in those days.

We congratulate the Professor on the research he has instituted and his results, which go far towards solving a great clinical problem.

REFERENCES.

"The Internal Secretion of the Pancreas," F. G. Banting and C. H. Best, *J. Lab. and Clin. Med.*, 1922, vii., 251.

"Pancreatic Extracts in the Treatment of Diabetes Mellitus," Banting, Best, Collip, Campbell and Fletcher, *J. Can. Med. Ass.*, 1922, xii., 141.

"Pancreatic Extracts," Banting and Best, *J. Lab. and Clin. Med.*, 1922, vii., 464.

"Preliminary Studies on the Physiological Effects of Insulin," Banting, Best, Collip, Macleod and Noble, *Tr. Roy. Soc. Canada*, Section v., 1922.

"Diabetes treated with Extract and by Grafts of Sheep's Pancreas," P. Watson-Williams, *Brit. M. J.*, 1894, ii., 1303; *Therap. Gaz.*, 1894, x., 649.

**Postgraduate Instruction.**

THE facilities provided by the University of Bristol for Postgraduate instruction have been much appreciated by local practitioners. The clinical assistantships at the Royal Infirmary and General Hospital have attracted a number of keen men. Courses of Postgraduate Demonstrations have been given to large classes in Trowbridge, Dorchester, Swindon, Hereford and Barnstaple during the last academic session.

For 1922–23 similar arrangements have been made for clinical work, and the Director of Postgraduate Studies in the University has had inquiries from India, Africa, Australia, as well as from all parts of England. Clinical assistants are appointed for one month, or thirty attendances, or for three months.

Postgraduate Demonstrations have been arranged for Bournemouth, Hereford and Barnstaple. These consist generally of a series of six classes; the subjects are suggested by local committees, and the meetings are held in a central hospital of the district.

In addition to these set courses the Director has been enabled to provide instruction in special subjects, both for routine work and as preparation for the higher examinations.

To take the results of the newer advances in Medicine and Surgery from a University centre into outlying areas was obviously a venture associated with many difficulties. But the enthusiasm, tact, and adaptability of those responsible for the demonstrations has surmounted these obstacles, while the interest and courteous consideration extended by the members of the classes has removed all doubts as to the expediency of continuance.

Requests for repeat courses have already been received from Trowbridge, Hereford, and Barnstaple, and this is the best gauge of their usefulness.

In addition to these special courses of lectures and clinical assistantships for short periods of study practitioners resident in Bristol and the immediate vicinity are invited to act as assistants (clinical) in the out-patient departments of the Royal Infirmary and General Hospital. These appointments are made for longer periods, for one year at the Infirmary and for six months at the Hospital. No fee is payable for the appointments, which are renewable. At the Royal Infirmary this system has been in vogue for many years, and the posts have been much sought after. The General Hospital has recently offered similar opportunities for keeping in touch with hospital work, and already there have been as many applications received as can be dealt with.

Those who are fortunate enough to live within easy reach of a teaching hospital have, in spite of the claims of their practices, found time to attend regularly over a number of years, rendering the most valuable help in the departments to which they are attached, and expressing themselves gratified by the advantages for continued study which they themselves obtain.

**Incorporation of the Medical School with the University.**

When Bristol University College was founded in 1877 some of the lecturers in the Medical School, and foremost among them Dr. Shingleton Smith, were anxious to see the School incorporated completely with the College. Various reasons, however, prevented this, and the School was merely affiliated. After a few years the preliminary subjects up to and including Anatomy and Physiology were handed over, together with the fees concerned, to University College. When the University was founded in 1909 the same plan was maintained.

At last, however, the Medical School has become entirely incorporated with the University. The clinical portion of the students' curriculum, which heretofore has been a proprietary affair of the honorary staffs of the Royal Infirmary and General Hospital, has been given over to the University and the students' fees have been surrendered to the University exchequer. The only private right the staffs retain is the privilege of introducing pupils to their hospital practice. This right is derived from a stipulation made between the original founders of the Bristol Royal Infirmary and the first surgeons elected to that institution, whereby the surgeons undertook to give gratuitous service on condition that they were allowed to introduce their apprentices to the wards of the Infirmary.

In virtue of this right the staffs are in the position to guarantee that University students can obtain the necessary clinical instruction. The surrender of the management of clinical teaching and the disposition of clinical fees was a big step which up to the present the staffs had not agreed to.

So long as the old arrangement lasted the Treasury, in making Government grants to the University, did not take into reckoning "clinical studies" in medicine as part of the functions of the University. In a recent communication to the University the Treasury Grants Committee remarked that they disapproved of "proprietary" medical schools and would make no grants in aid of them. The loss of grants which a continuance of the existing system entailed seemed disproportionate to any possible private advantage. These considerations convinced the staffs of the Infirmary and Hospital that a change was desirable. Now by a unanimous decision clinical teaching and clinical fees have been made over to the University of Bristol as from August, 1922. The University in return has arranged a scale of payments to clinical teachers which, although small, will have the advantage of not fluctuating with the number of students, and moreover is designed to remunerate teachers on the basis of work done, a plan which the old system did not by any means ensure.

One immediate innovation has been the appointment of three directors of clinical studies, whose chief duties will be to co-ordinate the teaching at the two institutions, and to organise on a coherent and continuous basis the clinical instruction of medical students. The directors appointed are Professor Edgeworth in medicine, Professor Hey Groves in surgery, and Professor Swayne in obstetrics and gynæcology. These newly-appointed directors start off with the good will of all their colleagues, and the scheme appears to have won the approbation of the students.

Thus the Bristol Medical School, the original foster-parent of University College, accepts wholly and unreservedly the University for its *Alma Mater*.

